# Beyond vascularization: aerobic fitness is associated with N-acetylaspartate and working memory

**DOI:** 10.1002/brb3.30

**Published:** 2012-01

**Authors:** Kirk I Erickson, Andrea M Weinstein, Bradley P Sutton, Ruchika Shaurya Prakash, Michelle W Voss, Laura Chaddock, Amanda N Szabo, Emily L Mailey, Siobhan M White, Thomas R Wojcicki, Edward McAuley, Arthur F Kramer

**Affiliations:** 1 Department of Psychology, University of PittsburghPittsburgh, PA; 2 Center for the Neural Basis of Cognition, University of PittsburghPittsburgh, PA; 3 Department of Bioengineering, University of IllinoisChampaign, IL; 4 Beckman Institute for Advanced Science and Technology, University of IllinoisChampaign, IL; 5 Department of Psychology, The Ohio State UniversityColumbus, OH; 6 Department of Psychology, University of IllinoisChampaign, IL; 7 Department of Kinesiology and Community Health, University of IllinoisChampaign, IL

**Keywords:** Aging, brain, exercise, fitness, human, N-acetylaspartate, working memory

## Abstract

Aerobic exercise is a promising form of prevention for cognitive decline; however, little is known about the molecular mechanisms by which exercise and fitness impacts the human brain. Several studies have postulated that increased regional brain volume and function are associated with aerobic fitness because of increased vascularization rather than increased neural tissue per se. We tested this position by examining the relationship between cardiorespiratory fitness and N-acetylaspartate (NAA) levels in the right frontal cortex using magnetic resonance spectroscopy. NAA is a nervous system specific metabolite found predominantly in cell bodies of neurons. We reasoned that if aerobic fitness was predominantly influencing the vasculature of the brain, then NAA levels should not vary as a function of aerobic fitness. However, if aerobic fitness influences the number or viability of neurons, then higher aerobic fitness levels might be associated with greater concentrations of NAA. We examined NAA levels, aerobic fitness, and cognitive performance in 137 older adults without cognitive impairment. Consistent with the latter hypothesis, we found that higher aerobic fitness levels offset an age-related decline in NAA. Furthermore, NAA mediated an association between fitness and backward digit span performance, suggesting that neuronal viability as measured by NAA is important in understanding fitness-related cognitive enhancement. Since NAA is found exclusively in neural tissue, our results indicate that the effect of fitness on the human brain extends beyond vascularization; aerobic fitness is associated with neuronal viability in the frontal cortex of older adults.

## Introduction

Given the projected increase in the proportion of older adults in the next 50 years, it is important to identify preventions and treatments for age-associated brain decay. Fortunately, aerobic exercise is a promising method to enhance neurocognitive function in older adults (Hillman et al. 2008; Erickson and Kramer 2009). Higher fit and physically active adults have greater brain volume in frontal and hippocampal regions than lesser fit and less active adults (Colcombe et al. 2003, 2006; Erickson et al. 2009, 2010, 2011; Honea et al. 2009). However, little is known about the molecular mechanisms contributing to enhanced volume. Several studies have postulated that increased regional brain volume is linked to increased vascularization, rather than the growth or expansion of neural tissue per se (e.g., dendritic spine density). Indeed, rodent studies have reported that exercise induces angiogenesis (Black et al. 1990; Kleim et al. 2004), and in humans fitness is associated with a greater number of small-caliber vessels in the brain (Bullitt et al. 2009). Therefore, it is likely that some of the volumetric differences related to fitness are due to increased vascularization.

Along these same lines, cardiorespiratory fitness and exercise are associated with enhanced task-induced and resting-state brain activity (Colcombe et al. 2004; Voss et al. 2010a; b), and cerebral blood flow and volume (Pereira et al. 2007; Burdette et al. 2010) as assessed by the blood oxygen level dependent (BOLD) response and arterial spin labeling (ASL). Since ASL and BOLD are inherently based on blood flow, it is possible that some of the functional magnetic resonance imaging (fMRI) effects are due to variation in vasculature. Several studies have argued against this, claiming that differences in brain function reflect the effect of fitness on the brain's processing capabilities and not just changes in the brain's circulatory system (Colcombe et al. 2004; Voss et al. 2010b). In line with this, rodent studies demonstrate that exercise affects cell proliferation and survival in the dentate gyrus (van Praag et al. 1999, 2005), dendritic complexity (Redila and Christie 2006), and molecules involved in learning and memory (Cotman and Berchtold 2002).

Because it is unclear the extent to which volumetric and fMRI results in humans are shaped by vascular differences, we examined a neurobiological measure that is unrelated to cerebral vasculature. Specifically, we examined whether higher fitness levels would be associated with greater concentrations of N-acetylaspartate (NAA). NAA is a nervous system specific metabolite (Nadler and Cooper 1972) found predominantly in cell bodies of neurons (Moffett et al. 1991). We reasoned that if aerobic fitness was predominantly influencing cerebral vasculature, NAA levels should not vary as a function of aerobic fitness. However, if aerobic fitness influences the number or viability of neurons, in addition to possibly influencing vasculature, then higher aerobic fitness levels should be associated with greater concentrations of NAA or offset any age-related reduction in NAA. Such a finding would support the argument that aerobic fitness influences neuronal viability in aged humans and provides additional insight about the mechanisms by which fitness enhances cognition.

## Methods

### Participants

One hundred thirty-seven community-dwelling participants (90 females; 47 males) between the ages of 58 and 80 years (mean age = 66.08; SD = 5.50 see Table 1) were recruited from Champaign-Urbana and east-central Illinois to participate in a randomized exercise intervention trial spanning one year. The results described in this study are limited to the baseline assessment of cardiorespiratory fitness and NAA. All participants were screened for cognitive impairment using the modified Mini-Mental Status Examination (Stern et al. 1987) and were excluded if the minimum score of 51 was not obtained (maximum score of 57). Additional inclusion criteria consisted of having normal or corrected to normal vision, absence of clinical depression as measured by the five-item Geriatric Depression Scale (>3; Sheikh and Yesavage 1986), and not very physically active as defined by participation in physical activity on two or fewer days of the week in the past six months. All participants met or surpassed safety criteria for participating in an MR study, including no history of head trauma, head or neck surgery, diabetes, neuropsychiatric or neurological conditions including brain tumors, or having any ferrous metallic implants that could cause injury due to the magnetic field. Individuals reporting the use of psychiatric or neurological medications were excluded from participation in the study. Finally, all participants provided physician's consent to engage in fitness testing and signed an informed consent approved by the University of Illinois.

**Table 1:** Participant characteristics.

Characteristic	Percent	Mean	SD	Range
Age (years)	—	66.08	5.50	58–80
Education (years)	—	15.85	2.90	8–24
Sex (% Female)	65.7	—	—	—
VO_2_ (mL/kg/min)	—	21.32	4.93	12.9–34.7
Weight (kg)	—	80.19	14.17	44–111
Creatine levels	—	8.68	1.38	5.61–11.7

### Aerobic fitness assessment

Aerobic fitness (VO_2_ peak) was assessed by graded maximal exercise testing on a motor-driven treadmill. The participant walked at a speed slightly faster than their normal walking pace (1.5–4.3 miles per hour), with increasing grade increments of 2% every other minute. An exercise test technologist continuously monitored measurements of oxygen uptake and heart rate while a cardiologist monitored EKG readings and a nurse assessed blood pressure. Oxygen uptake (VO_2_) was measured from expired air samples taken at 30-sec intervals until a maximal VO_2_ was attained or the test was terminated due to symptom limitation and/or volitional exhaustion. VO_2_ peak was defined as the highest recorded VO_2_ value when two of three criteria were satisfied: (1) a plateau in VO_2_ peak between two or more workloads; (2) a respiratory exchange ratio > 1.10; and (3) a heart rate equivalent to their age-predicted maximum (i.e., 220–age). VO_2_ peak scores are adjusted for weight, measured in units of milliliters per kilogram (mL/kg/min).

### Magnetic Resonance Spectroscopy (MRS) imaging protocol and data processing

**Figure 1:**
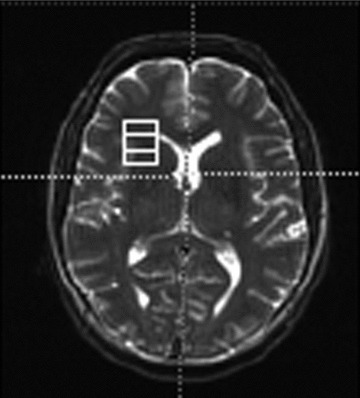
Placement of the 18 × 18 mm^2^ voxel in the frontal cortex. The voxel was positioned in the right frontal cortex so that it would contain insula and surrounding white matter. The voxel also included some tissue from caudate nucleus. Note: the image is in radiological coordinates.

A single 18-mm isotropic voxel was acquired in the right frontal cortex with a Repetition Time (TR) = 2000 ms and Echo Time (TE) = 30 ms using a spin echo single voxel spectroscopy sequence on a 3.0 T Siemens Allegra. The acquisition used water saturation and 128 averages of the spectroscopy acquisition with a 1200-Hz bandwidth. The single voxel was positioned so that it included tissue from gray and white matter, but did not include CSF. The voxel was positioned in the right frontal cortex, including areas in the inferior frontal gyrus, insula, and anterior portions of the basal ganglia (Fig. 1). We positioned this voxel in the frontal cortex based on research from previous studies demonstrating effects of exercise and fitness on prefrontal cortex volume and function (Erickson and Kramer 2009).

The spectroscopy data were processed in the Siemens Syngo 2004 spectroscopy analysis package (80333 Munich, Germany). The software provides peak fitting to common metabolites in proton spectroscopy with quantification of the integral of the spectrum for each peak. Values for NAA and creatine (Cr) were extracted from these data and used in subsequent analyses (see below).

### Behavioral tasks

#### Digit span task

The digit span subtest of the Wechsler Adult Intelligence Scale—Third Edition (Wechsler 1997) was administered to each participant approximately two weeks before the MRS session. The forward subtest of the digit span measures attentional capacity and short-term memory, whereas the backward subtest is often used as a measure of working memory capacity (Wechsler 1997). In this test, the experimenter read aloud a series of numbers at an interval of one number per second. After the experimenter completed the series of numbers, the participant orally repeated the same numbers either verbatim (forward span), or in the reverse order of presentation (backward span). The length of the to-be-remembered digits increased until the participant incorrectly responded to two presentations in a row or until the maximum span length was reached. The outcome measure of this test is span length, or the greatest number of digits correctly repeated (range of 0–9 digits for forward span; 0–8 digits for backward span). This test is used extensively throughout clinical and research studies and has high validity and reliability scores among healthy older adults (Wechsler 1997; Ryan and Ward 1999).

#### Spatial memory task

Participants performed a spatial memory paradigm that has been associated with aerobic fitness and hippocampal function in older adults (Erickson et al. 2009, 2011). First, a fixation crosshair appeared for 1 sec upon which participants were instructed to maintain fixation. Immediately following fixation, one, two, or three dots appeared at random locations for 500 msec. The dots disappeared for 3 sec, during which time participants were instructed to remember the dot locations. Next, a red dot appeared either in one of the same locations as the original targets or at a different location. Participants were instructed to respond as to whether the new dot was in the same or different location as any of the target dots. Reaction times (RTs) and accuracy (Acc) rates for each of the three dot conditions (1Dot, 2Dot, 3Dot) were analyzed in the current study. The spatial memory task used here is similar to the forward digit span task in that both tests assessed the maintenance of information in short-term memory storage, but the spatial memory task also requires relational memory and is therefore considered more dependent upon hippocampal functioning (Erickson et al. 2009, 2011). This is in contrast to the backwards digit span task, which is thought to be more dependent on prefrontal cortex functioning and is considered a more complex short-term working memory task than the digit forward condition.

### Statistical analyses

First, we examined the relationships between NAA, Cr, aerobic fitness, age, sex, years of education, digit span performance, and spatial memory performance by calculating Pearson correlation coefficients between all variables (see Table 2). It is customary for NAA levels to be examined relative to Cr levels (NAA:Cr); however, interpreting correlation and regression terms with ratio values is challenging because of possible variation in the denominator (Cr). Instead of using the NAA:Cr ratio, we chose to use Cr as a covariate of no interest in all multiple regression and mediation models described below in order to examine associations with NAA independent of any effects from Cr. The associations described below, however, did not change when using the NAA:Cr ratio as the variable of interest, indicating that associations with NAA and not Cr were driving the results. In addition, sex and education were correlated with several of the behavioral tasks and were therefore also used as covariates in all analyses (see Table 2).

**Table 2:** Pearson correlation coefficients between participant characteristics.

	Education	Sex	Creatine	Fitness	Age	NAA	F Span	B Span	1Dot RT	2Dot RT	3Dot RT	1Dot Acc	2Dot Acc
Education	—	—	—	—	—	—	—	—	—	—	—	—	—
Sex	0.257**	—	—	—	—	—	—	—	—	—	—	—	—
Creatine	−0.079	−0.310**	—	—	—	—	—	—	—	—	—	—	—
Fitness	0.316**	0.406**	0.058	—	—	—	—	—	—	—	—	—	—
Age	−0.073	0.057	−0.166†	−0.287**	—	—	—	—	—	—	—	—	—
NAA	0.033	−0.369**	0.467**	0.110	−0.155†	—	—	—	—	—	—	—	—
Forward span	0.065	0.121	0.048	0.124	−0.053	0.014	—	—	—	—	—	—	—
Backward span	0.223*	−0.055	−0.079	0.107	−0.098	−0.086	0.427**	—	—	—	—	—	—
1Dot RT	−0.004	−0.163†	−0.010	−0.284**	0.233**	0.082	0.015	−0.106	—	—	—	—	—
2Dot RT	−0.107	−0.223**	−0.025	−0.303**	0.228**	0.062	0.026	−0.164†	0.904**	—	—	—	—
3Dot RT	−0.115	−0.191*	0.003	−0.258**	0.214*	0.019	0.018	−0.119	0.861**	0.914**	—	—	—
1Dot Acc	0.014	−0.033	0.035	0.117	−0.258**	−0.005	0.130	0.175*	−0.244**	−0.217**	−0.100	—	—
2Dot Acc	0.050	0.095	−0.086	0.201*	−0.214*	−0.122	0.095	0.197*	−0.340**	−0.375**	−0.240**	0.782**	—
3Dot Acc	0.161†	0.098	0.019	0.313**	−0.345**	−0.027	0.207*	0.247**	−0.405**	−0.441**	−0.332**	0.745**	0.770**

RT, reaction time; Acc, accuracy.

† *P* < 0.10;

* *P* < 0.05;

** *P* < 0.01.

Age and VO_2_ peak were entered as predictor terms in multiple regression models, with NAA as the dependent variable of interest, to determine whether age and VO_2_ peak scores were associated with NAA levels. These models accounted for the variance associated with Cr, sex, and education. We also included an Age × Fitness interaction term in order to test whether higher fitness levels offset an age-related decline in NAA. Finally, we used multiple regression models to determine whether behavioral performance in both the spatial memory and digit span tasks was associated with NAA, VO_2_ peak, or Age, after controlling for Cr, sex, and education. Ordinary least squares linear regression was used for modeling the effects of NAA and fitness on spatial working memory. Since the digit span task variables of interest (span length) were categorical, we used a nonparametric within-subject randomization method to analyze digit span relationships with NAA and with fitness. In this analysis, 10,000 bootstrap samples were drawn with replacement to estimate a hypothetical probability distribution of NAA (or fitness) effects on digit span length. These distributions closely resembled normal distributions and were used as the comparison distribution for the current data, rather than comparing to a normal distribution. This method has been verified as a robust analysis method for nonparametric data (Chambers et al. 1983). Standardized β-values and *P*-values are reported for all regression analyses, along with *t*-values when relevant.

We also conducted an exploratory mediation model to determine whether NAA mediated a fitness–memory association. A mediation analysis is a hypothesis-driven model in which an independent variable is associated with a dependent variable indirectly through a mediating third variable. The traditional Baron and Kenny (1986) mediation method, requiring an initial association between the dependent variable and the independent variable, has been found to be an unnecessary requirement for mediation analyses (Baron and Kenny 1986; Gelfand et al. 2009; Zhao et al. 2010). The only necessity for mediation is that the indirect effect of the independent variable (e.g., fitness) through the mediator (e.g., NAA) on the dependent variable (e.g., digit span) be significant (Gelfand et al. 2009; Zhao et al. 2010). Mediation was determined by calculating the indirect mediation effect using bootstrapped sampling (Preacher and Hayes 2008). This method of mediation analysis is preferred over the Sobel test because the Sobel test assumes a parametric sampling distribution of the indirect effect. By using a bootstrapped regression analysis, nonparametric data does not violate any assumptions of the sampling distribution. In addition, bootstrapped mediation analysis has high power without increasing the Type 1 error rate (Preacher and Hayes 2008). In this analysis, 5,000 bootstrapped samples were drawn with replacement from the dataset, allowing for an estimation of the indirect mediation pathway (i.e., the pathway from aerobic fitness to NAA to cognitive performance). Indirect effects and 95% confidence intervals (CIs) were then computed from these results. All models controlled for sex, education, and Cr. An alpha level of *P* < 0.05 was used to determine significant effects.

## Results

### Aerobic fitness moderates an age-related decline in NAA

**Figure 2:**
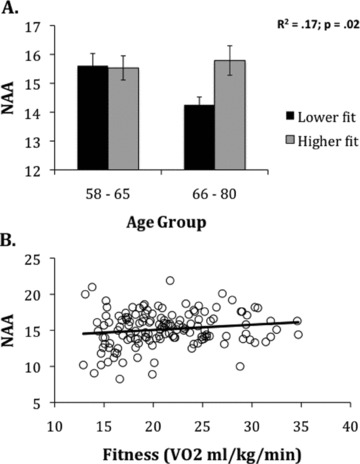

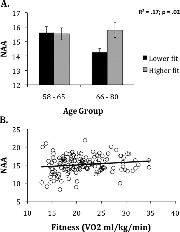
Relationship between N-acetylaspartate (NAA) and aerobic fitness levels. (**A**) Illustration of the relationship between fitness and age on NAA concentration. For illustration purposes, we plot Fitness and Age groups determined by a median split (median fitness score of 20.7 mL/kg/min; median age of 65 years). (**B**) Scatterplot of the relationship between fitness levels and NAA.

The primary aim of this study was to determine whether higher aerobic fitness levels were associated with higher levels of NAA and whether higher fitness levels offset an age-related loss of NAA. Consistent with our predictions, we found that older age was associated with lower NAA levels in the frontal cortex (β=−0.833; *t*=−2.542; *P*= 0.01) but that higher aerobic fitness levels offset the age-related decline in NAA, as demonstrated by a significant Age × Fitness interaction (β= 2.190; *t*= 2.586; *P*= .01). For further exploration of this interaction, we used the median values for fitness and age to divide the sample into a higher and lower fit groups (median fitness score of 20.7 mL/kg/min) and into older-old and younger-old groups (median age of 65 years). Consistent with the results from the regression, higher fitness levels offset an age-related reduction in NAA levels (Fig. 2A). Without the Age × Fitness interaction term in the model, there was also a main effect of fitness on NAA (β= 0.209; *t*= 2.314; *P* < 0.05) (Fig. 2B**)**. These results indicate that the effect of aerobic fitness on brain function in humans extends beyond vascularization of brain tissue and influences neuronal viability in the frontal cortex of aged adults.

### NAA and memory function

**Figure 3:**
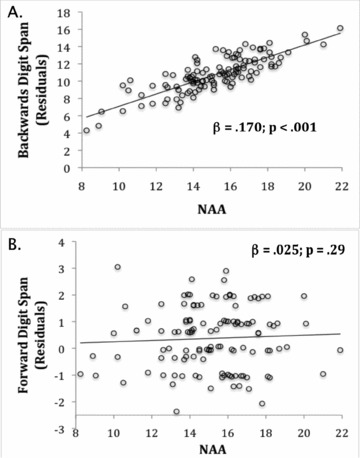
Relationship between NAA and digit span lengths. (**A**) Scatterplot of the linear relationship between NAA and the residuals of the backwards digit span bootstrap regression model after controlling for education, sex, Cr, and a quadratic trend in the span lengths. (**B**) Scatterplot of the null relationship between NAA and the residuals of the forward digit span bootstrap regression model after controlling for education, sex, and Cr.

We predicted that lower NAA levels would be associated with poorer cognitive function in older adults. Consistent with our prediction, lower NAA levels were associated with poorer working memory performance on the digit span backward task after controlling for the variance from education, sex, and Cr, as well as a quadratic trend in digit backward scores (β= 0.710; *P* < 0.001) in the bootstrap regression analysis (Fig. 3A). We also found that there was no relationship between NAA levels and forward digit span (β= 0.025; *P*= 0.29) when controlling for the variance from education, sex, and Cr in the bootstrap regression analysis (Fig. 3B). Furthermore, NAA levels were not predictive of response times or accuracy rates for any condition of the spatial memory task (all *P*s > 0.25) in linear regression models controlling for the variance from education, sex, and Cr. These results suggest that NAA in the frontal cortex specifically influences working memory capacity, but not short-term or relational memory as indexed by either forward digit span or spatial memory performance.

### Aerobic fitness and memory function

Aerobic fitness levels were not correlated with backward digit span lengths (*r*= 0.107; *P*= 0.23; see Table 2) or with forward digit span lengths (*r*= 0.124; *P*= 0.16). Using a bootstrap regression model controlling for age, sex, and education, the association between fitness and backward digit span performance was not significant (β= 0.03; *P*= 0.14), nor was the association between aerobic fitness and forward digit span (β= 0.02; *P*= 0.19).

Similar to prior results (Erickson et al. 2009), higher fitness levels were associated with faster RT in the one-item condition of the spatial memory task after controlling for the variance from age, sex, and years of education (β=−0.219; *t*=−2.154; *P* < 0.05). Higher fitness levels were also associated with higher accuracy rates for the most challenging three-item condition of the spatial memory task even after controlling for variance from age, sex, and years of education (β= 0.204; *t*= 2.064; *P* < 0.05).

### NAA mediates fitness and working memory relationship

Our final analysis tested whether NAA mediated the association between aerobic fitness and working memory when controlling for sex, education, and Cr. As described above, the only requirement for mediation is a significant indirect effect of the independent variable (e.g., fitness) through the mediator (e.g., NAA) on the dependent variable (e.g., digit span) (Gelfand et al. 2009; Zhao et al. 2010). Therefore, although we failed to find a significant association between fitness and memory in this sample after controlling for several covariates, we were still able to test the mediating effects of NAA on the fitness–cognition association. We found that NAA significantly mediated an association between cardiorespiratory fitness and backward digit span length (indirect effect =−0.011; 95% CI =−0.034 to −0.001). However, NAA mediation was not significant for the relationship between cardiorespiratory fitness and forward digit span length (indirect effect = 0.002; 95% CI: −0.009 to 0.017). Further, NAA did not mediate the association between fitness and spatial memory (all *P* > 0.05).

## Discussion

The extent to which results from brain volume (Bugg and Head in press; Colcombe et al. 2003, 2006; Erickson et al. 2007, 2009, 2010, 2011; Burns et al. 2008; Gordon et al. 2008; Honea et al. 2009; Peters et al. 2009; Chaddock et al. 2010a,b; Prakash et al. 2010; Rovio et al. 2010) and fMRI blood flow (Colcombe et al. 2004; Pereira et al. 2007; Prakash et al. 2007, 2011; Burdette et al. 2010; Rosano et al. 2010; Voss et al. 2010a,b; Smith et al. 2011) studies of aerobic fitness are dominated by differences in cerebral vasculature is unknown. On the one hand, it is clear that aerobic exercise increases the growth of new blood vessels in the brain (Rhyu et al. 2010). However, aerobic exercise also influences the proliferation of new neurons and increases the production of molecules secreted from neurons that are involved in learning and memory, such as brain-derived neurotrophic factor and insulin-like growth factor (Cotman and Berchtold 2002; Ding et al. 2006). Because of this, it is important to determine (a) whether aerobic fitness is associated with a nervous system specific measure in humans that is not confounded by differences in vascularization, and (b) whether a nervous system specific measure would be associated with better cognitive function. To this end, we measured the concentration of NAA, a metabolite found exclusively in the nervous system, and reasoned that if aerobic fitness predominantly influenced cerebral vasculature, then there should not be an association between aerobic fitness and NAA. On the other hand, if aerobic fitness influenced neuronal viability or metabolism, then higher aerobic fitness levels should be associated with greater concentrations of NAA or moderate an age-related decline in NAA.

Consistent with the latter prediction, we found that, in older adults, higher aerobic fitness levels offset an age-related decline in NAA. We also found that higher NAA levels were associated with greater working memory span, but not short-term attention or spatial memory, and that NAA mediates a fitness–working memory association. These results indicate that higher aerobic fitness levels are associated with greater neuronal viability, and that greater neuronal viability in the frontal cortex is selectively associated with elevated working memory function.

NAA is a metabolite found almost exclusively in the cell bodies of neurons where, in concert with astrocytes and oligodendrocytes, it plays a critical role in cellular metabolism and myelination (Moffett et al. 2007). NAA is essential for normal brain operation. This is evidenced by Canavan disease, an autosomal-recessive neurodegenerative mutation that deacetylates NAA, causing severe cognitive and psychomotor deficits, and death usually before 18 months of age (Matalon et al. 1988). Further, reduced NAA or NAA:Cr concentrations have been found in several neurodegenerative and neuropsychiatric diseases including Alzheimer's disease, stroke, multiple sclerosis, schizophrenia, epilepsy, bipolar disorder, and substance abuse disorder (see reviews by Moffett et al. 2007 and Ross and Sachdev 2004). Because of its nearly exclusive association with neurons, NAA is considered an in vivo measure of neuronal viability and metabolism (Nadler and Cooper 1972). The association between NAA and aerobic fitness, as well as the moderating effect of aerobic fitness on age-related losses of NAA, indicate that fitness should be conceived of as a viable method for enhancing neuronal viability in late adulthood. In fact, a recent small randomized, controlled trial found that patients with schizophrenia (*n*= 8) showed a 35% increase in NAA:Cr levels in a region of the left hippocampus after three months of aerobic exercise (Pajonk et al. 2010). In contrast, healthy control participants (*n*= 7) showed no change in NAA:Cr levels after the three-month trial. While these results are intriguing, especially for the patient group, the small sample size limits the generalizability of the results. A larger randomized controlled intervention for healthy older adults is needed to determine the direct link between exercise and neuronal integrity.

Our finding that aerobic fitness influences neuronal viability is consistent with a large body of research on the effect of exercise in rodents. Voluntary wheel-running increases the production of new neurons in the dentate gyrus of the hippocampus (van Praag et al., 1999, 2005), increases dendritic complexity (Redila and Christie 2006), and enhances the production and secretion of molecules involved in augmenting learning and memory (Cotman and Berchtold 2002; Kramer et al. 2006). Human neuroimaging studies have found greater brain volume in higher fit individuals (Erickson et al. 2009, 2011), and increased blood volume and activation during attentional control and memory tasks (Pereira et al. 2007; Colcombe et al. 2004; Prakash et al. 2011). Although the results that we describe here do not eliminate the possibility that fitness-induced vascularization is playing a role in prior volumetric and fMRI studies, our results do indicate that cerebral vasculature is not the only explanation for fitness-related augmentation of brain and cognitive function.

Our results probably do not reflect neurogenesis in the frontal cortex, but instead probably reflect increased neuronal metabolism, increased neuron size and viability, or elevated neuronal signaling. In any case, as stated above, increased neuronal viability in the frontal cortex in relation to aerobic fitness demonstrates that the effects of exercise extend beyond a simple “brain circulation” hypothesis. Nonetheless, measures of increased vascularization and neuronal viability are closely coupled and are difficult constructs to completely separate. It is likely that greater aerobic fitness is associated with increased vascularization of the frontal cortex, which is contributing to increased neuronal viability.

There are several important limitations of our study. First, the cross-sectional nature of the design leaves open the possibility that an unmeasured third variable covaries with aerobic fitness levels and that fitness is not the fundamental factor contributing to these results. It will be important for the results from the randomized controlled intervention to examine whether NAA concentrations can be altered during the course of an exercise regimen. Second, cross-sectional and observational studies often suffer from multicollinearity among the assessed variables. Our study was not immune from multicollinearity problems and, because of this, the regression coefficients might be inflated. While we examined standard error values in order to assess possible inflation effects, results from the randomized intervention should help even further to reduce these possible problems in multicollinearity and allow for statistical modeling that account for high correlations among measured variables. Third, although we used two different memory paradigms in this study, it will be important for future studies to test the association between NAA in the frontal cortex and other types of memory, including episodic, procedural, and semantic memory. Fourth, even though all participants were carefully screened for psychiatric and neurological conditions, it is possible that preclinical neuropathology was affecting brain volume, NAA levels, and/or cognitive function. Finally, scanner limitations precluded our ability to obtain NAA concentrations from more than a single voxel. Because of this, we decided to focus on NAA concentrations in the frontal cortex, where fitness effects have been documented in humans (Colcombe et al. 2003, 2004, 2006; Erickson and Kramer 2009). Recent developments in MR spectroscopy allow for multiple voxel acquisition so that NAA can be obtained from several brain regions in a single acquisition. Acquisition of NAA from several different brain regions, including the hippocampus, will be important to determine the degree to which fitness and exercise have specific or general effects on the neurobiology of the human brain.

In sum, we demonstrate, in a large sample of well-characterized and healthy older adults, that higher aerobic fitness levels ameliorate an age-related decline in NAA concentrations in the frontal cortex, and that higher NAA concentrations mediate the association between aerobic fitness and working memory span. These results indicate that higher aerobic fitness levels are effective at moderating reductions in neuronal viability that occur in late life. Since NAA is found exclusively in the nervous system, our results indicate that the effect of fitness on the human brain extends beyond vascularization; aerobic fitness influences neuronal viability in the frontal cortex of older adults.
